# Classifying Residues in Mechanically Stable and Unstable Substructures Based on a Protein Sequence: The Case Study of the DnaK Hsp70 Chaperone

**DOI:** 10.3390/nano11092198

**Published:** 2021-08-26

**Authors:** Michal Gala, Gabriel Žoldák

**Affiliations:** 1 Department of Biophysics, Faculty of Science, P. J. Šafárik University, Jesena 5, 040 01 Košice, Slovakia; michal.gala@student.upjs.sk; 2 Center for Interdisciplinary Biosciences, Technology and Innovation Park, P. J. Šafárik University, Trieda SNP 1, 040 11 Košice, Slovakia

**Keywords:** Hsp70, substructures, physico-chemical features, machine learning

## Abstract

Artificial proteins can be constructed from stable substructures, whose stability is encoded in their protein sequence. Identifying stable protein substructures experimentally is the only available option at the moment because no suitable method exists to extract this information from a protein sequence. In previous research, we examined the mechanics of *E. coli* Hsp70 and found four mechanically stable (S class) and three unstable substructures (U class). Of the total 603 residues in the folded domains of Hsp70, 234 residues belong to one of four mechanically stable substructures, and 369 residues belong to one of three unstable substructures. Here our goal is to develop a machine learning model to categorize Hsp70 residues using sequence information. We applied three supervised methods: logistic regression (LR), random forest, and support vector machine. The LR method showed the highest accuracy, 0.925, to predict the correct class of a particular residue only when context-dependent physico-chemical features were included. The cross-validation of the LR model yielded a prediction accuracy of 0.879 and revealed that most of the misclassified residues lie at the borders between substructures. We foresee machine learning models being used to identify stable substructures as candidates for building blocks to engineer new proteins.

## 1. Introduction

Stable protein super-assemblies have recently been designed and engineered to form functional nanodevices such as nano-cages for therapeutic applications [1,2,3,4]. To increase the number and the complexity of these super-assemblies, mechanically stable building blocks are prerequisites. The stability and structure of the building blocks are fully encoded in their protein sequence. However, short sequences can form different structures of different stabilities that are impacted by the presence of other folded substructures, which suggests a long-range contextual dependence.

Protein folding and stability have been studied for decades, and many crucial theoretical concepts and principles have been revealed [5]. Some of the challenges in protein research have remained, e.g., it is currently not possible to reliably determine whether substructures of folded proteins will assume a folded form or not when they have been isolated. For example, a substructure derived from villin headpiece wild-type is unstable and exists as a random-coil structure, whereas the N68A/K70M variant forms a stable α-helical substructure [6,7].

In this study, we focus on the dichotomy between mechanically stable and unstable substructures, which we recently discovered using mechanical force experiments on a multi-domain Hsp70 protein. In general, predicting the mechanical properties of proteins and the mechanical stability of protein substructures is very challenging due to the absence of a validated conceptual framework. While three-dimensional structures can now be accurately predicted [8,9,10,11], the most precious information about the stability of individual substructures has yet to be revealed. In the critical assessment of protein structure (CASP) competition, the main goal is to determine the structure. However, at the moment, the stability of the substructures can only be predicted with great difficulty, if at all [12]. Due to high cooperativity between interacting substructures, it is unclear whether a particular substructure is stable in isolation.

Hence, some of the challenges are inherent to the cooperative nature of substructures and to the mechanical anisotropy of proteins, which means that the mechanical properties of proteins are highly dependent on pulling orientation [13,14,15,16]. Single-molecule studies showed that during the unfolding of large proteins, folded protein substructures are disrupted in distinct and well-defined steps [16,17,18,19,20,21,22,23,24,25]. The high reproducibility and specificity of these microscopic unfolding steps indicates a significant level of cooperativity. After the unfolding of large proteins, single-molecule force spectroscopy identified several mechanically stable substructures as folding intermediates or partially folded well-defined structures that are stable even in the absence of other folded substructures in the rest of the protein as exemplified by a large number of intermediates in adenylate kinase [18], DnaK [17,19], Hsp90 [23,24,25], and calmoduline [26]. Based on detailed mechanical studies, structural borders of stable intermediates were identified, and, in some cases, the existence of the partially folded substructures was confirmed independently in isolation and through biochemical characterization [20]. Such shorter substructures were found to be autonomously folded domains and their properties were verified by traditional assays [7,20].

**Figure 1:**
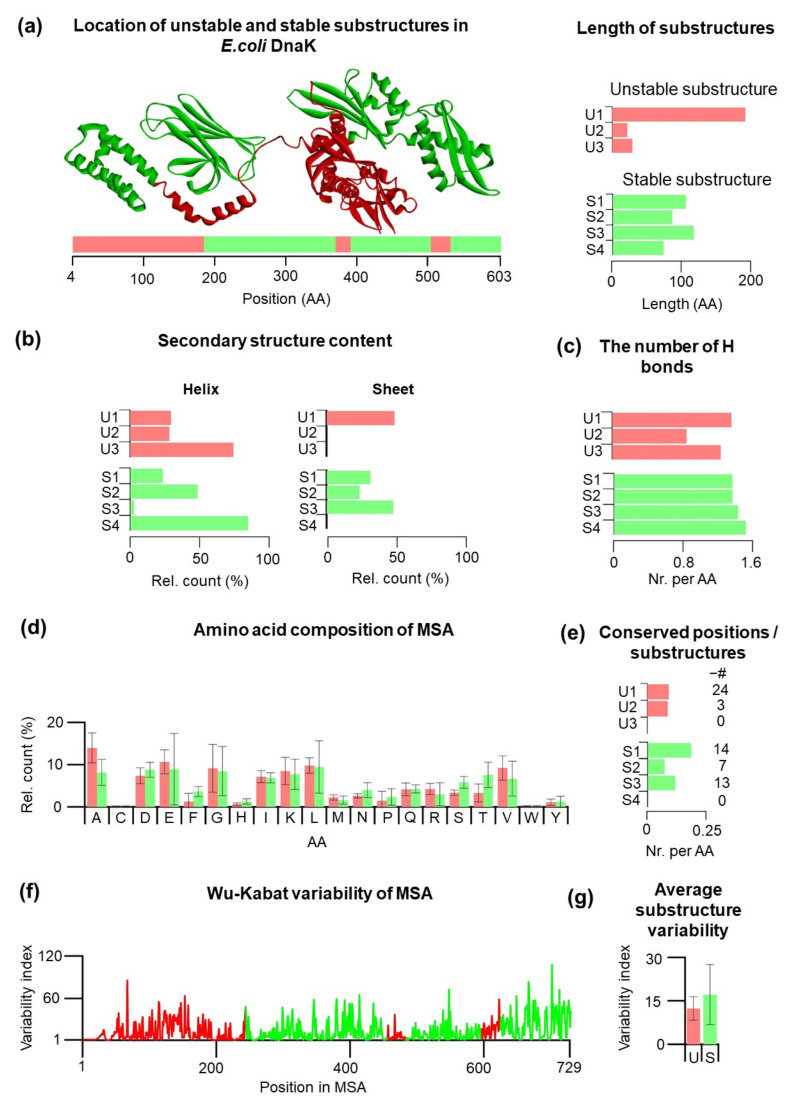
Structural and sequence characterization of Hsp70s and their substructures belonging to U (red) and S (green) classes (see also Supplementary Figure S3). (**a**) The 3D structure and the length of substructures are shown in the closed form (2KHO) of *E. coli*. The protein consists of three mechanically unstable and four mechanically stable substructures. Substructure S1 is split into two parts by the inserted domain—the substructure S2. (**b**) Secondary structure content of α-helix and β-sheet of S1–S4 and U1–U3 substructures for *E. coli* DnaK. (**c**) The number of hydrogen bonds per amino acid for U/S substructures. (**d**) Amino acid composition of 205 Hsp70 sequences. The error bars showed variability of the amino acid composition of Hsp70s. (**e**) Conserved positions per amino acid in substructures and absolute numbers of conserved positions for U/S substructures from MSA. (**f**) Wu–Kabat variability of 205 Hsp70 sequences obtained from MSA. (**g**) Averaged variability of residues in U/S substructures obtained from the MSA of 205 Hsp70 sequences. There are no significant differences in variabilities between classes.

Recently, we conducted a series of single-molecule nanomechanical studies on DnaK, the Hsp70 chaperone from *E. coli* [16,17,19,20]. Using laser optical tweezers for mechanical studies, we examined mechanical properties of both Hsp70 domains: the nucleotide-binding domain (NBD) and the substrate-binding domain (SBD). In these experiments, we found that several substructures can fold even in the absence of other folded substructures. The NBD consists of two stable substructures (S1, S2, Figure 1a)—lobe IIa, a discontinuous domain that can fold only after lobe IIb, which can fold very quickly. The SBD consists of two stable substructures (S3, S4, Figure 1a) that belong to a C-terminal helical bundle and a functional β-core (see also Supplementary Figure S1). These four Hsp70 substructures we labeled as mechanically stable substructures (S class) to indicate their significant mechanical stability and these substructures can be classified as autonomously folding units as well. The stable substructures S2–S4 are separated by three unstable substructures (U1–U3, U class, Figure 1a). Some of the residues lying at the S/U borders, so they are encompassed by the different class residues.

Classifying residues that belong to stable protein substructures and hence identifying them using sequence information would be highly useful when screening protein databases for stable building blocks. Along this line, our group has identified a stable substructure and ATP-binding mini-domain that can be easily combined with a subdomain from a yeast mitochondrial homolog, which yields new chimeric and functional fully folded proteins [20].

Here we ask whether amino acid residues that are located in mechanically stable or mechanically unstable substructures can be distinguished based on their physico-chemical properties. While the physical theories cannot predict stability from sequence information, a heuristic approach is to apply machine learning methods to generate a model that can predict with high accuracy. Even though we have successfully developed machine learning models for Hsp70 protein, there are no limitations to apply our conceptual framework to any other protein. Now, the major limitation is the availability of experimental data on internal protein nanomechanics. As the experimental work on protein mechanics continues, several high-quality experimental datasets can then be used to develop efficient and accurate machine learning models that reliably predict stable substructures from the sequence information only.

This paper is divided as follows: First, we present a post hoc structural analysis of *E. coli* Hsp70 followed by phylogenetic analysis of 205 Hsp70s. Of these 205, 183 sequences are bacterial DnaK (including nine paralogs), 12 Hsp70 are from Archea and 10 from Eukaryota. Second, we focus on unsupervised and supervised machine learning methods. To this end, 28 physicochemical features, as well as one-hot encoding, were used to find informative projections in the principal component analysis (PCA). Substructures were classified using linear discriminant analysis (LDA). In the first naive approach, we assumed context-free features and were not able to develop a successful learning model for classification. To improve our model, a sequence context was included by applying the moving average algorithm, which uses pre-defined window sizes. Then, LDA and PCA methods were better able to distinguish between two classes of residues located in either mechanically stable or unstable substructures. In particular, LDA at relatively large window sizes was partially successful at distinguishing and classifying the residues into S/U classes. However, we found that the classification was not robust enough. For more accurate S/U class prediction, three machine learning models were used: logistic regression (LR), random forest (RF), and support vector machine (SVM). All these methods were able to identify and distinguish residues located in stable and unstable substructures at good accuracy; the logistic regression model performed best, with an accuracy of 0.925 (before the cross-validation procedure). In the next step, the cross-validation procedure of the logistic regression model was conducted, and a final accuracy of 0.879 was obtained. We found that the most of the misclassified residues are located at the borders of the S/U substructure class.

## 2. Materials and Methods

For the analysis, *E. coli* Hsp70 sequence ID sp|P0A6Y8, as well as the set of 205 Hsp70 sequences, was taken from Uniprot/Swissprot database (https://www.uniprot.org/). One hundred eighty-three of these sequences are bacterial DnaK, including nine paralogs, twelve Hsp70 from Archea, and ten are eukaryotic Hsp70. Structural analysis was conducted on the closed form of *E. coli* Hsp 70 (accession PDB code: 2KHO) [27] using Discovery Studio (BIOVIA, Dassault Systèmes, Discovery Studio, San Diego: Dassault Systèmes, 2019). Using this program, we calculated secondary structure content, average number, and average lengths of intramolecular H-bonds. Sequence alignment was generated using MEGA X [28] by applying the MUSCLE algorithm [29]. The following settings were used: gap open: −2.90, gap extension: 0, hydrophobicity multiplier: 1.2, and clustering method: UPGMA. The sequence identity matrix was calculated after sequence alignment using the program BioEdit [30]. According to the manual, for each pair of sequences, score values are calculated as indicated: (i) all positions are pairwise compared, one at a time, (ii) all ‘gap’ or place-holding characters are treated as a gap, (iii) positions where both sequences have a gap do not contribute (they are not an identity, they do not exist), (iv) positions where there is a residue in one sequence and a gap in the other do count as a mismatch, (v) reported number represents the ratio of identities to the length of the longer of the two sequences after positions, where both sequences contain a gap, are removed. 

Wu–Kabat variability values describe the susceptibility of an amino acid position to be replaced during evolution [31]. It highlights stretches of accentuated amino acid variation. The value of Wu–Kabat variability is computed using the following equation:

(1) $$\frac{N\times k}{n}\;$$

where *N* is the number of sequences in the alignment, *k* is the number of different amino acids at a given position, and *n* is the frequency of the most common amino acid at that position. Wu–Kabat variability values were calculated using Protein Variability Server (http://imed.med.ucm.es/PVS/). All data analysis, calculations, normalizations, moving averages, PCA, LDA, and implementation of the supervised models were performed in the KNIME data analytics tool (https://www.knime.com/). Feature pairwise correlation was calculated as Pearson correlation coefficient. For data normalization, we used Z-score normalization, which means that values in each column are Gaussian distributed, i.e., the mean value is 0 and the standard deviation is 1. The formula for Z-score normalization is below:

(2) $$\frac{value-\mu }{\sigma }$$

The moving average was calculated by the center Gaussian moving average method. Here *v_n_* is the value in the *n*-th row of the data table in the selected column, and *k* is the window size.

(3) $$Center\;gaussian=\overset{n}{\underset{i=0\ldots k-1}{\sum }}\left(\frac{i,\left(k-1\right)}{2,stdev}\right)\times v_{n}+\left(\frac{i-\left(k-1\right)}{2}\right)$$

For the Gaussian weighted moving average, individual values are weighted according to their position in a given window:

(4) $$stdev=\frac{k-1}{4}$$

and the weighting factor:

(5) $$gauss\left(i,mean,stdev\right)=\exp^{\frac{\left(-0.5\right)\times {\left(i-mean\right)}^{2}}{stdev^{2}}}$$

Attention was paid to the feature values at the beginning and at the end of the Hsp70 sequence. The first and last values were omitted so that the central value of the moving average was calculated with the full size of the window. The window size was varied from 1 to 31 amino acids. For the PC analysis, informative projections were calculated using Orange [32]. In this approach, for every 2D projection 10 nearest neighbors were identified. Hence, combinations of pairs of features are found. Next, counting of features with identical labels provides the score of the projection. The following machine learning supervised methods were used: logistic regression, random forest, and support vector machine. In all methods, the values of the attributes were normalized. Regarding the training and testing set: for each window size, we selected 40 amino acids from the beginning and 40 amino acids from the end of the Hsp70 sequence for the testing. Hence, the first 40 amino acids belong to the class of unstable substructures (U1), and the other 40 amino acids belong to the stable substructures S4. The other positions were used as a training set. In the selection of residues for the training set, we selected residues that are not affected by the residues used for testing purposes. Because of significant window size in some cases, training residues may contribute to the features of nearby residues, and hence they can affect each other. To this end, special care was devoted to select positions used for training, which depends on the size of the window used for moving average. Feature selection was performed using a forward feature selection algorithm. It is an iterative approach, which starts with no feature selected. In each iteration, the feature that improves the model the most is added to the feature set. Using the final selected feature set, parameter optimization was then conducted using at least 1000 iterations, and, in the case of SVM, the brute force method was used. In this method, all possible parameter combinations of the given learning model (given the intervals and step sizes) are evaluated, and the best (the highest accuracy) is returned. For LR and RF, the number of iterations was optimized as well. For SVM, three parameters were optimized: power, bias, and gamma for polynomial kernel type. Cross-validation ran in 10-folds, and partitions were sampled randomly. For these partitions, the class distribution has been preserved. A synthetic set of artificial physicochemical features was created by randomized mixing of values for individual amino acids only within a given feature (see also Supplementary Materials). There was no exchange of the amino acid values between different features.

## 3. Results

### 3.1. Analysis of the Stable and Unstable Substructures of Hsp70

Hsp70 consists of 638 amino acid residues that belong to either mechanically stable or unstable protein substructures (Figure 1a)—based on our previous research [16,17,19,20]. For our analysis, we excluded residues 604–638 because they belong to a low-complexity intrinsically disorder sequence, which differs dramatically from the folded substructures. The strategies for the identification of low-complexity disordered regions have been published [33]. In total, there are three unstable substructures: U1 = lobe I (res. 1–185, NBD), U2 = the linker (res. 371–392), and U3 = helix A and part of helix B (res. 506–533), and there are four stable substructures: S1= lobe IIa (res. 186–228 + 312–371, NBD), S2 = IIb (res. 229–311, NBD), S3 = β-1-8 (res. 393–505, SBD), and S4 = α-helices B–E (534–603). When we compare the sizes, the size of unstable substructures is highly variable and varies from 21 to 185 residues, while the size of stable substructures is more homogeneous and varies from 70 to 113 amino acid residues. On average, unstable substructures are shorter than stable substructures.

The mechanical stability of stable and unstable substructures may depend on the secondary structure content, and therefore, we analyzed the secondary structure content of the substructures in their full-length folded form (Figure 1b). In this full-length form, the first initial residues are not resolved; therefore, our analysis started at residue 4. The analysis of the individual substructures shows that stable substructures have a preference for β-sheets, while only one out of three unstable substructures contains a significant amount of β-sheets. Thus, secondary structure preferences can be different between different classes of U/S substructures, which might be reflected by the different number of hydrogen bonds.

To follow up on this idea, we have included the analysis of intramolecular hydrogen bonds in individual substructures (Supplementary Figure S2). The number of hydrogen bonds per amino acid is slightly lower for U substructures compared to stable substructures. The lowest number of hydrogen bonds per residue are found for U2 (0.857), followed by U3 (1.25). On the other hand, S4 showed the highest number of hydrogen bonds per residue (1.514), likely the result of the helical characters (84.29%) of this substructure. In principle, the mechanical properties of Hsp70 substructures can have different evolutionary constraints, which can be deduced from phylogenetic analysis (see also Supplementary Materials). We collected 205 sequences of Hsp70 mostly from eubacteria and performed multiple sequence alignment (MSA). The MSA enabled us to have a deeper look at the average amino acid composition of the substructures, the number of conserved residues within each substructure, and amino acid variability. In the first step, the average amino acid composition of the substructures was analyzed (Figure 1d). There are subtle differences in amino acid composition; for example, Ala is slightly more presented in unstable substructures (13.94%) than stable substructures (8.15%). On the other hand, it appears that stable substructures consist slightly more Thr (7.56%) compared to unstable substructures (3.29%). This is consistent with a statistically higher number of intramolecular hydrogen bonds per residues for S substructures since Thr contains the OH group that participates in hydrogen bonding. Next, for each substructure, we analyzed the number of conserved regions in different substructures (Figure 1e). Because substructures have a different number of amino acid residues, we calculated the number of conserved residues per single amino acid residue (CSR/residue). The total number of CSR is indicated in parentheses (Figure 1e). Substructures U3 and S4 do not have any conserved positions. Substructure S1 has the largest proportion of conserved positions per amino acid (0.18), and also it has the most conserved positions within the S class overall (14 positions). In the U class, U1 has the largest proportion of the conserved positions per amino acid (0.117) (24 positions).

On average, stable substructures have a slightly higher number of CSR, with S1 substructure as the highest value for CSR/residue. On the other hand, S4 and U3 substructures, both located in the SBD, do not have any CSR.

A slightly higher CSR/residue may indicate that stable substructures can have additional constraints; therefore, we extended our analysis by calculating Wu–Kabat variability for each residue (Figure 1f). The analysis indicates a similarity between profiles of the U1 substructures and S1 + S2. There is no clear indication that U class have higher variability compared to S class. Interestingly, the S4 substructure shows large variability and large oscillations between the variability of the individual residues. Average variability of the stable and unstable Hsp70 substructures (Figure 1g) points out a large spread of the residue variability index for S substructures. It should be emphasized that the analysis mentioned above is based on a posteriori knowledge of the mechanical behavior of Hsp70 substructures and no prediction model can be develop yet.

### 3.2. Sequence Context is Crucial for the Categorization of Residues within Mechanically Stable and Unstable Substructures

In the last part, post hoc analysis was performed to find structural and phylogenetic features by which the individual substructure classes can be distinguished. The approach identified possible differences between stable and unstable substructures and, hence, at least in theory, differentiation between substructure classes can be learned based on these properties. Moreover, we further collected another 28 different physico-chemical features of all proteinogenic amino acids from the list (Table 1).

**Figure 2:**
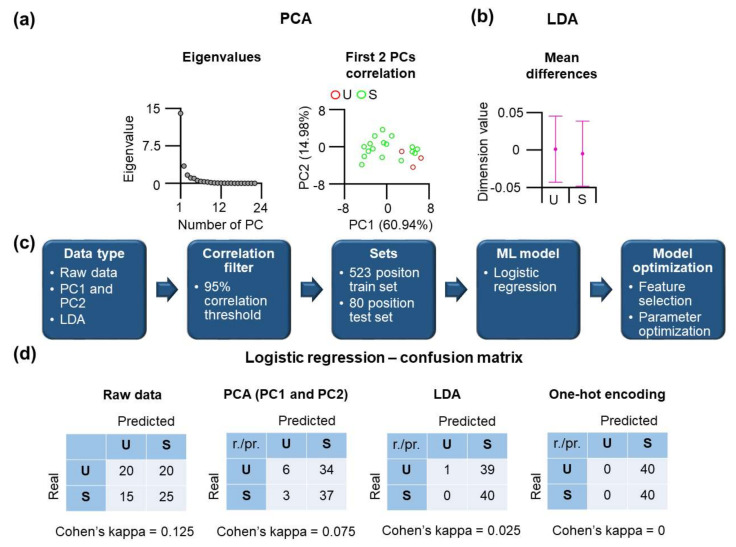
Application of PCA, LDA, and logistic regression methods to classify residues at 1 aa window size. (**a**) The PCA method. Eigenvalues of all PCs (left) and the plot (right) of PC1 (60.94% of total variance) versus PC2 (14.98% of the total variance). (**b**) Mean differences of dimension values between U and S classes were obtained by the LDA method. (**c**) The workflow for predictive machine learning model using logistic regression method at 1 aa window size. (**d**) Confusion matrix of logistic regression prediction using four different data types (raw data, first two PCs, LDA data, one-hot encoding data).

First, we applied principal component analysis (PCA) to determine whether we can spot differences between classes (Figure 2a). The Scree plot shows that the first components already contain most of the variance. Plotting of PC1 (60.94%) and PC2 (14.98%) shows many overlapping values for stable and unstable substructures with no apparent separation between the categories. There is a minor number of points that U and S substructures differ from each other. To analyze whether it is possible to find a linear combination of features that separates two mechanically distinct substructures, we performed a linear discrimination analysis, LDA (Figure 2b). LDA showed only poor performance; however, slight differences exist that may be utilized for learning approaches consistent with PCA. Therefore, we applied the logistic regression method to our original data in our next endeavor and to transformed data from PCA and LDA. The workflow of the analysis is shown in Figure 2c.

Before data were used as an input, a correlation filter was applied (see Methods). For training, all available distributed positions were used for training and. The logistic regression (LR) was then used primarily because other ML methods yielded similar results, and as we will demonstrate later, LR has the best performance among other ML methods such as random forest and support vector machine. To maximize the accuracy of the LR method, several rounds of parameter optimization were conducted. The results of this learning method are shown in the form of a confusion matrix (Figure 2d), including the corresponding Cohen’s kappa value. Cohen’s kappa shows very low values for all cases, below 0.125 that indicates poor performance. In the same line, the confusion matrix results show the failure of logistic regression learning to distinguish residues located in mechanically distinct substructures.

In a previous part, we used physico-chemical features of amino acids, which may not be optimal for machine learning because different amino acids may have similar physico-chemical features. We, therefore, applied an approach where each amino acid is uniquely described by a matrix consisting of 20 columns that have value 1 for identity or zero for else. The results of this so-called one-hot encoding are shown in Figure 2d. However, even for this encoding, there is no learning possible, and Cohen’s kappa value drops to 0. Our findings indicate that LR fails to learn using context-free features, which means that the developed machine learning model cannot distinguish the residues located in U or S substructures. To improve the models, we decided to include the local context of amino acids. The reason is that residues within the individual substructures differ by their local sequence and consequently by the physico-chemical context. This context can be approximated by, e.g., using an average moving procedure to calculate a new set of features that reflect nearby residues.

### 3.3. Context-dependent Features can be Obtained by the Application of Moving Average

**Figure 3:**
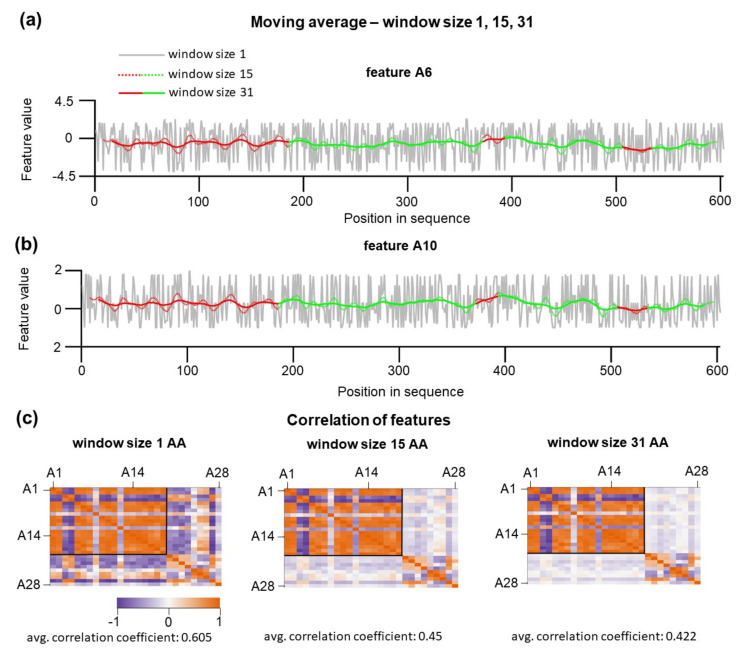
Effect of three different moving average window sizes (1 amino acid (aa), 15 aa, 31 aa) on feature values. (**a**,**b**) Effect of window size 1 aa (grey line), window size 15 aa (red/green dotted line), and window size 31 aa (red/green full line) on feature A6 and feature A10 values along the *E. coli* DnaK sequence. (**c**) Heat maps showing correlations between features as a function of different window sizes. Features A20 to A28 characterize the physio-chemical state of a single aa. Their values and pairwise correlations do not change with window size. Average correlation coefficients are shown bellows the heat maps. These correlations are the largest between the features at 1 window size, and the pairwise correlations decrease as the window size increases.

In our initial naïve approach, we considered only individual amino acid properties; within a polypeptide chain, the physical properties of the individual residues are affected by the nearby amino acids. Hence, the local chemical context of residues can play a crucial role in defining that a given substructure will be mechanically stable or not. To include the local sequence context, a new set of features were re-calculated using the moving average (MA) procedure that provides a measure of the average local properties for a custom-defined window’s size. In this algorithm, features of the residues encompassing the central position were weighted by the central Gaussian method. In the Gaussian method, nearby amino acids contribute less than distal amino acids (see Methods). The window size defines the cut-off over which residues are assumed to have zero contribution. MA procedure was applied for features A1–A19 because the calculated averages have a plausible physical meaning as local sequence-dependent hydrophobicity. It is physically feasible that for individual substructures, such calculated hydrophobicity is context-dependent and not a sole property of individual amino acids. Other features (A20–A28) such as polarity, refractivity, and mutability were not averaged and considered as not strongly dependent on a local sequence. As expected, moving average has a significant effect on the feature variations (Figure 3a). Large oscillations of feature values along the sequence are smoothed, and each of the positions starts to be context-dependent. Smoothing and averaging of the A6 and A10 feature values are dependent on window size. The effect of window sizes on feature values is shown in Figure 3a,b, where the window size was set to 1, 15, and 31 amino acids. In all our further analyses, we limited the maximum value for the window size to 31. This upper limit is given by the amino acid length limitation of the sequence. A more extended window size would result in completely neglecting information about the positions in this shortest substructure. At such large window sizes, residues located in U3 would not be considered for training or testing purposes. For testing at the window size 1, N-terminal 40 positions (U class) and 40 C-terminal positions (S class) were used. For testing at larger window sizes, we kept the total number of trained positions constant. However, as the window size increased, the moving average was not calculated for the positions close to the end. To solve this missing end issue, we shifted the positions for the training set toward protein sequence by the value n-1, where n is the window size. One crucial point is that hypothetically increasing the window size leads to a smoothing of individual differences along the sequence and, as a consequence, one would expect that such smoothing would, even more, reduce the information content of how individual amino acids relocated in U or S substructures.

However, this is not true. In particular, because the application of moving average has a profound effect on the overall pairwise correlation between features. Pearsons correlations between 28 features are shown in Figure 3c. By increasing window size, the overall correlation among features decreases from 0.61 for 1 aa to 0.42 for 31 aa. The decrease in the correlation between features results from unequal transformations of features; A20–A28 did not undergo moving average transformations. The observed loss of feature correlations can be explained by residues within distinct clusters while hydrophobic clusters potentiate values of A1–A20 features, the presence of the chemically identical amino acid at different positions encompassed by the cluster of polar amino acid will lead to diminishing correlation with A20–A28 features.

Having a new set of context-dependent features, we applied the PCA method to question whether MA at a specific window size can provide a possible strategy to categorize residues within S and U substructures (see also Supplementary Figure S4). Our workflow consists of the utilization of a new set of amino acid features. Again, a correlation filter was applied, and highly correlated (r2 more than 0.95) were removed (Supplementary Table S1, Figure S5). It is important to emphasize that for different window sizes, different features were selected. After the selection, data were normalized, and PCA was applied, which yields principal components that can have different informative values. After the calculation, we found very that the first PCs was not informative in respect class separation. Informative PC projections were evaluated by the calculating the separation values for U/S classes.

**Figure 4:**
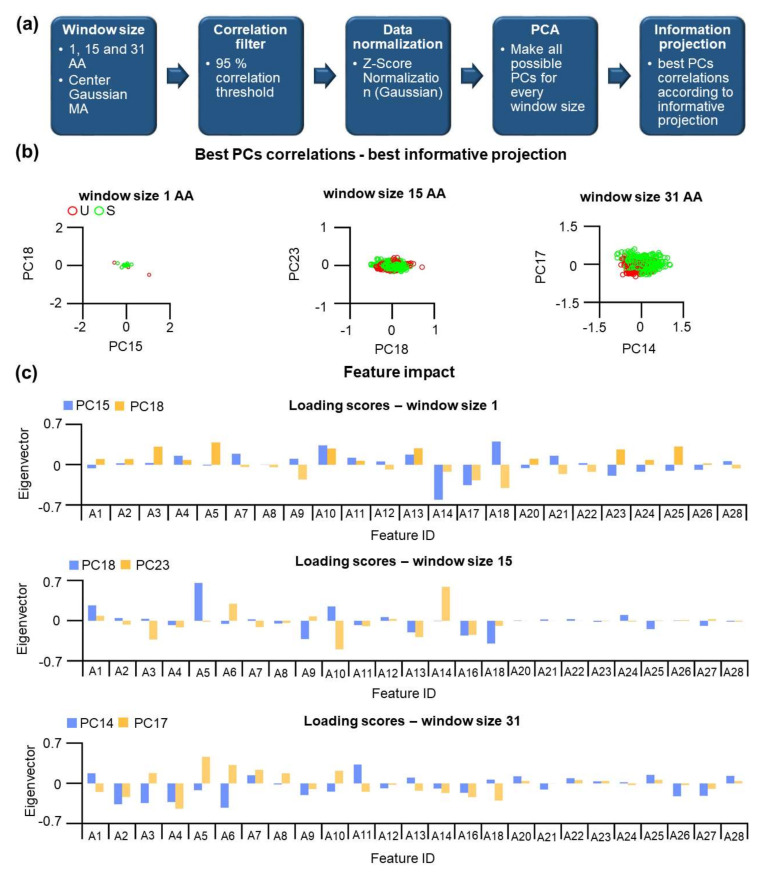
The application of the PCA method on data using feature values at different window sizes. (**a**) The workflow for the PCA method. (**b**) Best informative projections for classification of U/S at different window sizes. (**c**) Corresponding loading scores (from PCs in **b**) for all features used for the PCA method. Different features were used due to the fact that some of them did not pass through the correlation filter (see **a**).

We found that higher PCs were more informative than the first PCs, even though the first PCs cover a significant amount of the data variance. For example, for window size 15, we found that PC18 and PC23 are the most informative projections, while for window size 31, the projection of PC14 vs. PC17 provides the best separation Figure 4b. For U and S substructure classes, clusters are visible. However, a significant overlap exists. Notably, depending on the size of the window used for MA, different PCs are selected, and we asked whether these different PC share similar features. Loading scores for the PC components are shown in Figure 4c, and they quantify how the individual features are represented in different PCs. Interestingly, even after MA application, features A20–A28 show a progressively smaller but distinct contribution to the PCs with the best informative projections. The amplitude of the loading score indicates their minor importance for the separation between categories.

In summary, the PCA method shows low performance in categorizing positions for the U and S substructure classes, and no clear cluster separation can be found. Using MA and algorithm for the finding of informative projections, the performance of PCA is slightly improved but still inefficient for a precise categorization. In the next step, supervised machine learning methods are applied.

### 3.4. Linear Discriminant Analysis can be used to Find Differences between the Positions within U and S Categories

In our previous effort, the performance of the unsupervised PCA method can be improved by the finding of the informative projection. The result indicates that a small but significant difference between positions in U and S substructure classes exists. Hence, we decided to apply LDA to maximize differences between categories.

**Figure 5:**
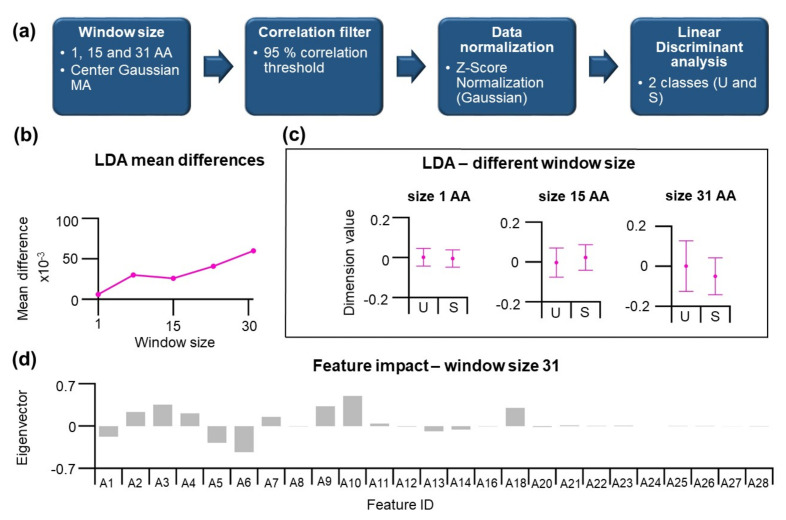
The application of the LDA method on data using feature values at different window sizes. (**a**) Workflow for the LDA method. (**b**) LDA mean differences of U and S classes—dimensions values as a function of window size. LDA difference increases with window size. (**c**) A closer look at dimension values at 1, 15, and 31 window size. (**d**) Eigenvectors values for the features at 31 aa window size.

The workflow consists of identical steps as described before (Figure 5a). Here, two categories of residues located in U and S substructures are described by 28 features. The LDA results in n-1 dimension reduction, where n is the number categories, and the data are represented by a 1D data array that can be used to classify the given residue to U or S substructure classes. The application of moving average has increased the differences between categories. The performance of LDA can be visualized by comparing differences in dimension values for U and S classes, which is shown in Figure 5c. As mentioned earlier, in the absence of MA, the performance is inferior. This increase shows a roughly linear dependence on the window size (Figure 5b). For window size 15 and 31 amino acids, the difference in dimension values for U and S increases, indicating a better classification of the data. A closer look at eigenvectors for LDA at 31 aa window size is shown in Figure 5d. Here, the features A20–A28 show values close to zero, indicating their weak contribution, consistent with loading scores of the informative projections from PCA. The effect of moving average is a clear benefit for the LDA, and many MA-treated features show high magnitudes of eigenvector. Among the highest absolute values belong the features A6 and A10.

### 3.5. Supervised Machine Learning Models

Based on the improvement of the data classification using the LDA method, we employed other supervised machine learning models: logistic regression (LR), random forest (RF), and support vector machine (SVM).

First, we divided our dataset into a training and a testing set. We took 40 amino acids located at the very N-terminal part (U class) and 40 amino acids from C-terminal part (S class) for testing. For training, positions were taken at a minimal distance from the border of the testing positions; the distance is equal to the size of the window used for the MA. This distance was necessary to eliminate overlap between the values in the train/test sets and ensure that used positions are genuinely independent.

**Figure 6:**
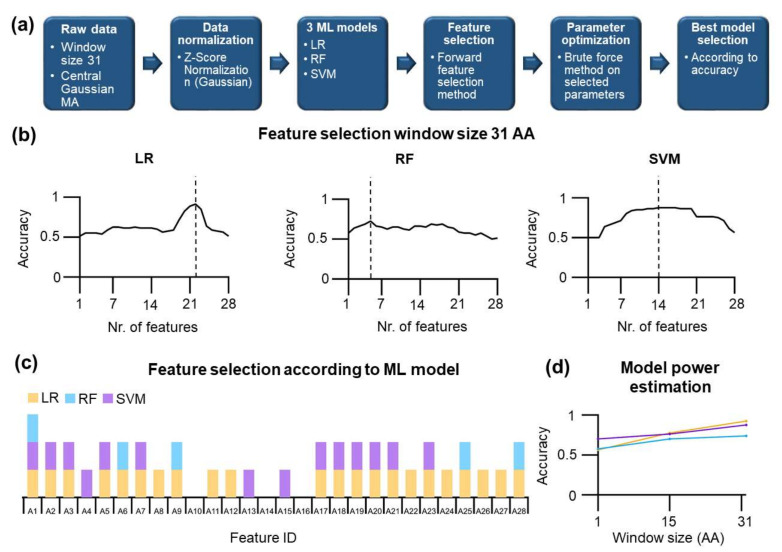
The application of supervised ML methods (random forest—RF, support vector machine—SVM, logistic regression—LR) to data at 31 aa window size. (**a**) Workflow for the ML methods. (**b**) The number of features, inputs for the learning, as a function of the accuracy for U/S prediction of three ML algorithms using the forward feature selection method. Based on the accuracy criterion, LR was the best ML method (0.913). Here, the methods are before the parameter optimization. (**c**) Selected features for the ML models were chosen based on their usage for the models with the highest accuracy. Only feature A1 was used in all three models. (**d**) Models accuracy after features selections and parameter optimizations at window sizes 1, 15, and 31 aa window size MA. The LR model showed the best accuracy (0.925).

A naive application of ML models resulted in a coin-flip performance at any window size and model used, i.e., the accuracy of ca. 0.5–0.56. A more elaborate strategy was needed to maximize the accuracy, including the feature selection step followed by the brute force parameter optimization. Specifically, we used the forward feature selection method, and consistently with our previous results, no improvement could be found for context-free features (see also Supplementary Figure S6) and the application of moving average improved the accuracy. For window size 31, the results of feature selections are shown in Figure 6b. For LR, the maximum accuracy of 0.925 is found using 22 features (see Figure 6b, and after parameter optimization 6d). For RF, 0.738 is the maximum accuracy found using five features. For the SVM, 14 features are the optimum number that yields an accuracy of 0.875. First, not all features were used for learning by different ML methods; some features are shared among learning methods, and some features are unique for a given method (Figure 6c). 

Only one feature, A1, is presented in all optimized ML methods. Two learning models shared 14 out of 28 features. Three features were not used in any learning model (A10, A14, A16). 

A high accuracy may be due to overfitting. To assess whether overfitting can be an issue in our analysis, we calculated Cohen’s kappa, which was reasonably high for all methods: 0.85 for LR, 0.75 for SVM, and 0.48 for RF. High Cohen’s kappa values for LR and SVM indicate that we can rule out the overfitting. The high accuracy of different ML methods for a window size of 31 was further analyzed by reducing the window size and performing feature selection/parameter optimization rounds. As expected, reducing window size led to a decrease in the accuracy of the models in all methods (Figure 6d). Overall, LR slightly outperforms all of the other methods. Therefore, we continue further with a validation of the LR method. To validate the LR model, we designed a so-called shuffling test. In this test, we took all 22 features identified in the feature selection process for the LR model and shuffled the values for individual amino acids within each feature. Hence, here we would like to estimate how the physico-chemical properties of amino acids contribute to the performance of the LR model (Table 2). Namely, amino acids with similar chemical structures do have similar values of their features. The shuffling of the values naturally results in a decreased overall correlation between them—from 0.422 to 0.156 for shuffled data at window size 31. Hence, features used in the shuffled test are much more unique and more diverse. Using shuffled features, the accuracy of the LR model decreases to 0.3875 and Cohen´s kappa decreases to −0.225. Such values indicate the very poor performance of the LR model with shuffled features.

### 3.6. Cross-Validation of the LR Method

**Figure 7:**
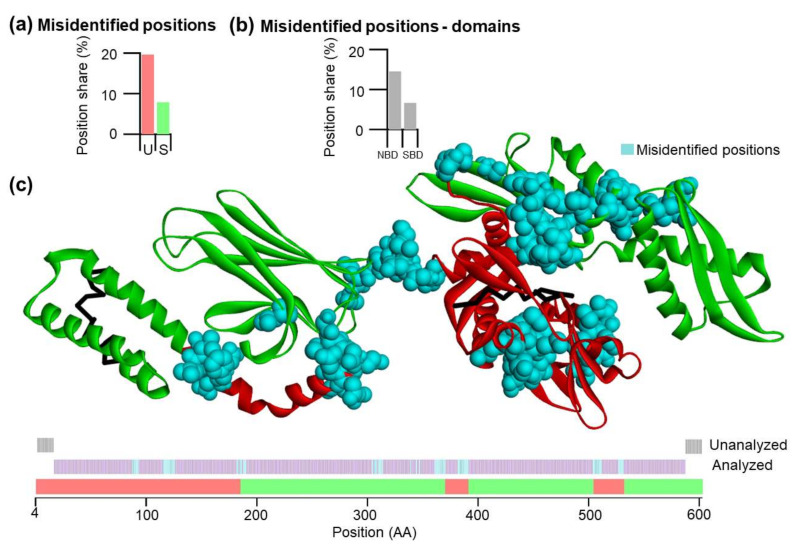
Cross-validation of the LR model on residues in the U/S substructure classes of *E. coli* DnaK. (**a**) The percentage of misidentified positions according to total numbers of the analyzed positions in U/S classes. Approximately more than 2/3 of all misidentified positions are U-class positions. (**b**) The percentage of misidentified positions in NBD and SBD. It shows that ca. 50% of misidentified positions are in the NBD. (**c**) The LR model accuracy was visualized on the 3D closed structure (2KHO) of DnaK *E. coli*. Non-analyzed positions are in black, and misidentified positions are turquoise. Misidentified positions are mainly localized on borders between substructures of different classes.

The LR model with 22 features at a window size of 31 amino acids showed the highest accuracy (0.925), and therefore the LR method was used for cross-validation (see also Supplementary Table S2). In the cross-validation approach, the size of the test set was 57–58 positions, which were distributed equally between U and S categories. The procedure was repeated 10 times (10-folds) and we found that the standard deviation of k-fold errors is 3.63% which indicates a high robostness. After cross-validation, the LR model reached an accuracy of 0.879 and Cohen´s kappa of 0.741 (Table 3). The precision of the residue classification was 0.863 and 0.884. The analysis of misidentified positions showed that most of the misidentified residues were located between the borders of U and S substructures (Figure 7). In general, there are more misidentified positions in U substructures compared to positions within S substructures, and more misidentified positions are in the nucleotide-binding domain of DnaK. In this approach, caution is needed because test/train sets are not explicitly separated.

We applied cross-validation to other machine learning methods (see Section 3.5). Even though these approaches had slightly weaker performance, yet they support our overall concept of learning. Cross-validation statistics are summarized in Supplementary Table S2. Overall, and for both SVM and RF methods, the residues of the S class showed a higher F-score indicating a higher accuracy of classification.

## 4. Discussion

Machine learning models have been used to solve biological problems such as predicting solubility of proteins, targeting subcellular localizations, folding and more [58,59,60,61,62,63]. In this paper, we develop a machine learning model that utilizes protein sequence information, which can classify residues in mechanically stable and unstable substructures. The best performance was achieved with a logistic regression, which showed the highest accuracy, 0.922, and a high Cohen’s kappa parameter, 0.85. Two factors were essential for the development of a successful model.

The first factor is to use physico-chemical parameters of the individual amino acids. We were not able to develop an accurate machine learning model employing one-hot encoding, which indicates that the physico-chemical information encoded in amino acids is crucial. The most significant difference between one-hot encoding versus physico-chemical parameters is the values for individual amino acids. In one-hot encoding, the values for individual amino acids are binary: 0 or 1. However, the physico-chemical values for individual amino acids have a broader range of values. Additionally, the values are not randomly distributed; instead, chemically similar amino acids tend to be grouped together and have similar physico-chemical values, which results in grouping amino acids as polar, charged, hydrophobic, etc. In one-hot encoding, such grouping is not present. Note that grouping chemically similar amino acids is also observable in evolutionary relations between amino acids, such as in BLOSUM substitutions matrices. These relations indicate that an amino acid can easily replace a chemically similar amino acid in a protein sequence. Successful development of the machine learning model demonstrates that a significant amount of information is captured in the similarities of the physico-chemical parameters of amino acids. To test these conclusions, we generated artificial physico-chemical parameters, where the values for the individual amino acids were shuffled and hence different amino acids, some of them highly chemically dissimilar, were grouped. Using these artificial parameters, we were not able to develop an accurate learning model, which also rules out overfitting due to a large number of features.

The second factor is to include the local sequence context in the learning, which can be realized by including the moving average algorithm. In this algorithm, increasing window sizes improved the performance of our machine learning model. The moving average window generates locally averaged values and simultaneously provides a unique value specific to a given residue in the polypeptide chain. Increasing window sizes thus progressively generates unique physico-chemical values that facilitate learning. This approach, however, has a limitation: the number of non-overlapping positions used for learning and training sets decreases proportionally. Classifying residues in the short substructures has an additional intrinsic problem that large window sizes locally average over different substructures eventually over various classes of substructures. We used the moving average algorithm in unsupervised and supervised methods. In PCA, as an unsupervised method, the most informative projections (the largest difference between U/S classes) were found at very high PC numbers (PC14 and PC17—in the case of window size 31), which suggests that the difference between U/S classes is quite small and presents an only tiny fraction of the total variance (0.4659% for PC14, and 0.152% for PC17). The progress in the learning is clearly seen in the LDA (Figure 5); the increase in the difference between the residues in the S and U class has non-trivial dependence likely due to compensation of the features. Here, context-dependent features showed the highest impact. Next, three different machine learning methods, logistic regression, random forest, and support vector machine, were applied and processed through the several rounds of the feature selection and optimization. From the three ML methods, logistic regression showed the best performance and an accuracy of 0.92. Again, increasing the window size of the moving average up to 30 residues has improved the accuracy of the predictions.

Next, using the cross-validation procedure, we observed that there are several and systematic misclassified residues. In particular, misclassified residues around the positions 182–183 can be due to the original assignment of the domain borders; exact borders are difficult to identify. Hence, misclassified residues must not be truly misclassified and can be that they are correctly predicted by the model and wrongly assigned by the experimental results. Cross-validation showed that positions between borders are problematic, possibly due to the moving average method that includes positions from the S class and vice versa. We conclude that the U/S classes can be distinguished by their contextual features.

In the SBD, more misclassified residues are likely due to higher variability, as shown by Wu–Kabat variability analysis. Phylogenetic analysis of Hsp70s showed an increased Wu–Kabat variability of the SBD compared to the NBD. High variability of the SBD is independent of the U/S class found in *E. coli*, which indicates that either (i) mechanical stability of the given substructure is not evolutionarily conserved for other Hsp70, or (ii) if it is conserved, then there are no significant sequence restrictions on sequence space for U/S class. Similarly, for the NBD, there are no differences in variability profiles of the substructures of U/S class. The absence of evolutionary conservation of the mechanically stable substructures can be rationalized by the greater importance of the function over the mechanical stability of individual substructures, given the overall structure is stable enough. The absence of sequence space restrictions for stable substructures appears less probable.

Here we show that we can classify residues to U/S class at a reasonable accuracy; however, the model’s capacity is limited to Hsp70, and the number of predicted substructures is low. Predicting mechanically stable structures would be highly beneficial for protein engineering and design because it would identify stable building blocks needed for complex structures.

The robustness of our concept is reflected by the results of cross-validation and by comparison of the different methods used: logistic regression, random forest, and support vector machine (Supplementary Tables S3–S6). We would like to emphasize that in the presented case study, the number of mechanically stable substructures is low, and hence the algorithm will show a much weaker performance in other cases. The major weakness of our approach is to take a constant window size as a proxy for a local context, which is due to a small testing set. Using adaptive window size—as a part of a reinforced learning algorithm—may provide a more physically realistic approximation of the local effects. Hence, our approach provides a conceptual framework based on single-molecule mechanics data, which can be further improved by extending the testing set.

In summary, predicting the mechanical stability of proteins is challenging due to an insufficient amount of experimental testing data. To speed up the progress, one possibility would be applying a more targeted approach—massively parallel design synthesis and testing strategy—to develop robust machine learning models to improve the accuracy and efficiency of predictions of stable substructures [2].

## Figures and Tables

**Table 1:** List of features with their respective ID.

ID	Name of Feature
A1	Hydropathicity [34]
A2	Hydrophobicity (delta G1/2 cal) [35]
A3	Hydrophobicity (free energy of transfer to surface in kcal/mole) [36]
A4	Hydrophobicity scale based on the free energy of transfer (kcal/mole) [37]
A5	Hydrophobicity scale (contact energy derived from 3D data [38]
A6	Hydrophobicity scale (pi-r) [39]
A7	Hydration potential (kcal/mole) at 25 °C [40]
A8	Hydrophilicity [41]
A9	Average surrounding hydrophobicity [42]
A10	Hydrophobicity scale (pi-r) [43]
A11	Membrane buried helix parameter [44]
A12	Antigenicity value X 10 [45]
A13	Hydrophobicity scale (Contribution to the stability of globular proteins) [46]
A14	Free energy of transfer from inside to the outside of a globular protein [47]
A15	The proportion of residues 95% buried (in 12 proteins) [48]
A16	Mean fractional area loss (f) [average area buried/standard state area] [49]
A17	Hydrophobicity of physiological L-alpha amino acids [50]
A18	Optimized matching hydrophobicity [51]
A19	Normalized consensus hydrophobicity scale [52]
A20	Average flexibility index [53]
A21	The atomic weight ratio [54]
A22	Polarity [55]
A23	Molar fraction (%) of 3220 accessible residues [47]
A24	Refractivity [56]
A25	Average area buried on transfer from standard state to folded protein [49]
A26	Bulkiness [55]
A27	Polarity [54]
A28	Relative mutability of amino acids (Ala = 100) [57]

**Table 2:** Comparison of LR model on real versus synthetic features (see also Supplementary Figure S7).

Set (Window Size 31 AA)	Category	Recall	Precision	F-Measure	Accuracy	Cohen’s Kappa
Real features	U	0.85	1	0.9189	-	-
	S	1	0.8696	0.9302	-	-
	Overall	-	-	-	0.925	0.85
Synthetic features	U	0.25	0.3448	0.2899	-	-
	S	0.525	0.4118	0.4615	-	-
	Overall	-	-	-	0.3875	−0.225

**Table 3:** The statistic of the LR model after cross-validation.

Set (Window Size 31 AA)	Category	Recall	Precision	F-Measure	Accuracy	Cohen’s Kappa
All positions	U	0.8037	0.8713	0.8361	-	-
	S	0.9268	0.8844	0.9051	-	-
	Overall	-	-	-	0.8797	0.7414

## Supplementary Materials

The following are available online at https://www.mdpi.com/article/10.3390/nano11092198/s1, Figure S1: DnaK subdomains. Figure S2: Average length of hydrogen bonds in DnaK from *E. coli* (pdb: 2KHO). Figure S3: Pairwise sequence identity of Hsp70s based on multiple sequence alignment. Figure S4: PCA eigenvalues at different window sizes. Figure S5: Correlation filter for 28 features. Figure S6: Forward feature selection used on three different ML methods at window size 1 AA and 15 AA. Figure S7: Heat maps of feature correlations for real feature values and for synthetic feature values at window sizes 1 AA and 31 AA respectively. Table S1: Correlation filter at different window sizes. Table S2: 10-folds Cross-validation of LR on whole data set. Table S3: 10-folds Cross-validation of SVM on whole data set. Table S4: Statistics of SVM model on whole data set. Table S5: 10-folds Cross-validation of RF on whole data set. Table S6: Statistics of RF model on whole data set.
