# Supplementary material for: Classifying Residues in Mechanically Stable and Unstable Substructures Based on a Protein Sequence: The Case Study of the DnaK Hsp70 Chaperone
Source: Nanomaterials (Basel). 2021 Aug 26;11(9):2198. doi: 10.3390/nano11092198 (PMC8467864; doi:10.3390/nano11092198)
Supplement: Supplementary file 1 [file nanomaterials-11-02198-s001.zip › nanomaterials-1328693-supplementary.pdf]

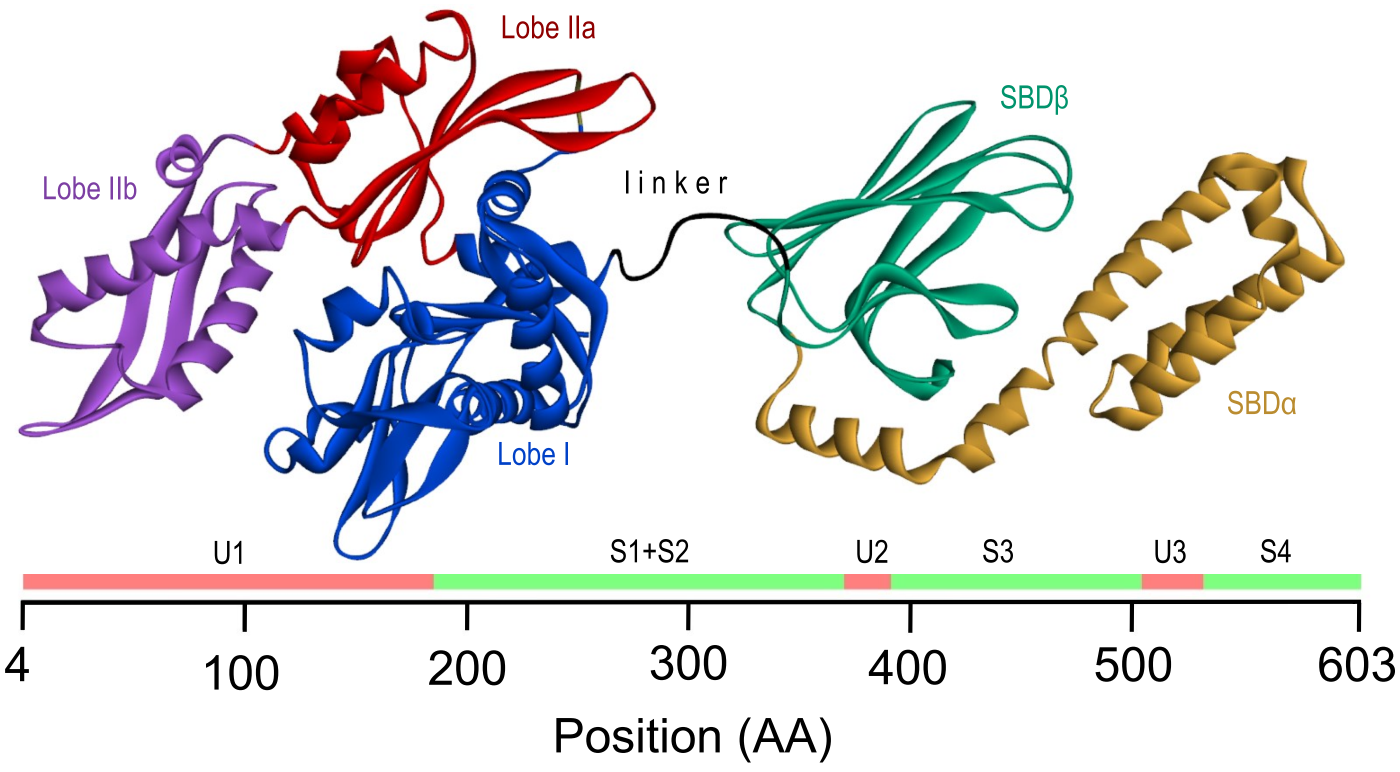

Figure S1. DnaK subdomains

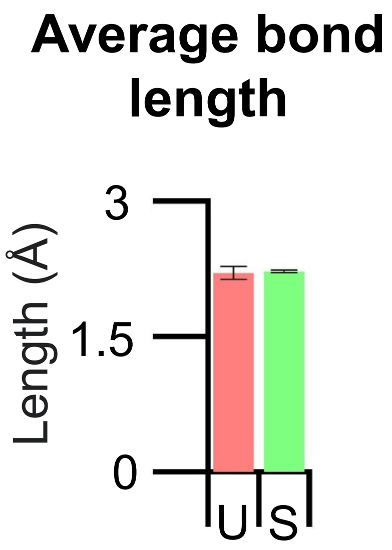

Figure S2. Average length of hydrogen bonds in DnaK from *E.coli* (pdb: 2KHO). The average length of bonds in unstable class is 2.204 A and in stable classes is 2.224 A. In general, error bars show there are no differences between this two classes.

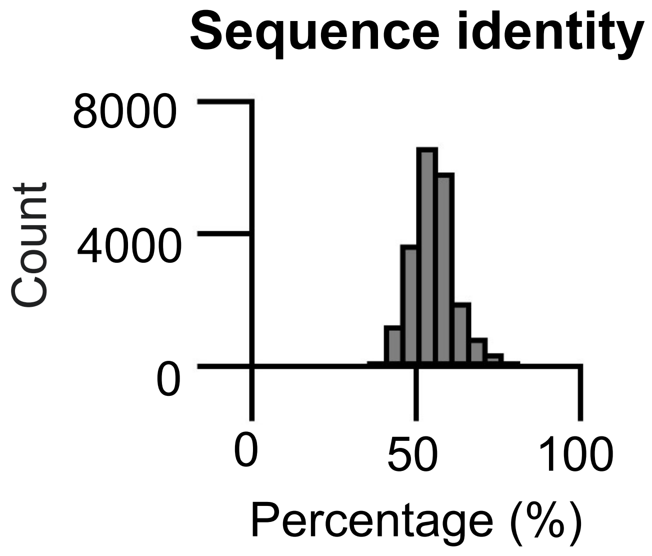

Figure S3. Pairwise sequence identity of Hsp70s based on multiple sequence alignment. Sequence identities of Hsp70s are in the interval between 35 and 80 %. Most identities (6635 out of 20910) fall into the 55% - 50% interval.

PCA

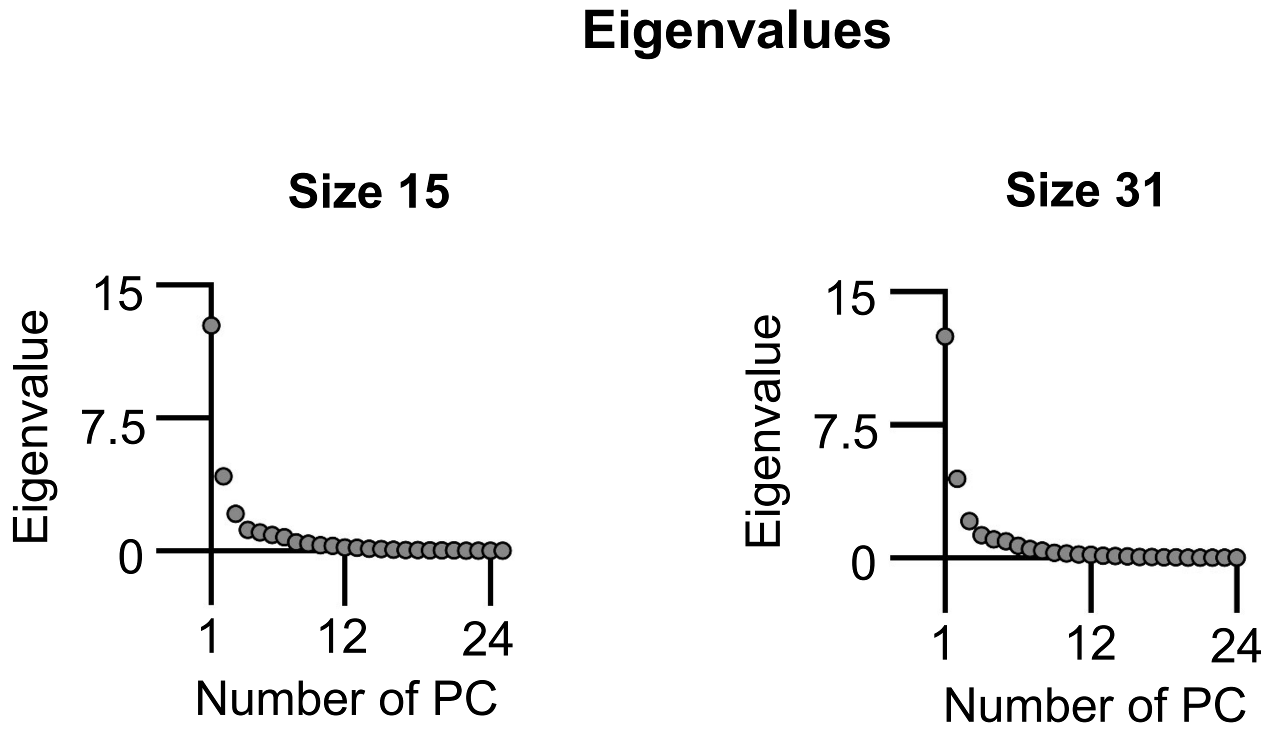

Figure S4. PCA eigenvalues at different window sizes. Eigenvalues of all PCs calculated at window size 15 and 31.

A **Correlation filter**

Table S1: Correlation filter at different window sizes.

Window size	Number of features
[redacted]	[redacted]
15	25
[redacted]	[redacted]

B **Correlation threshold filter**

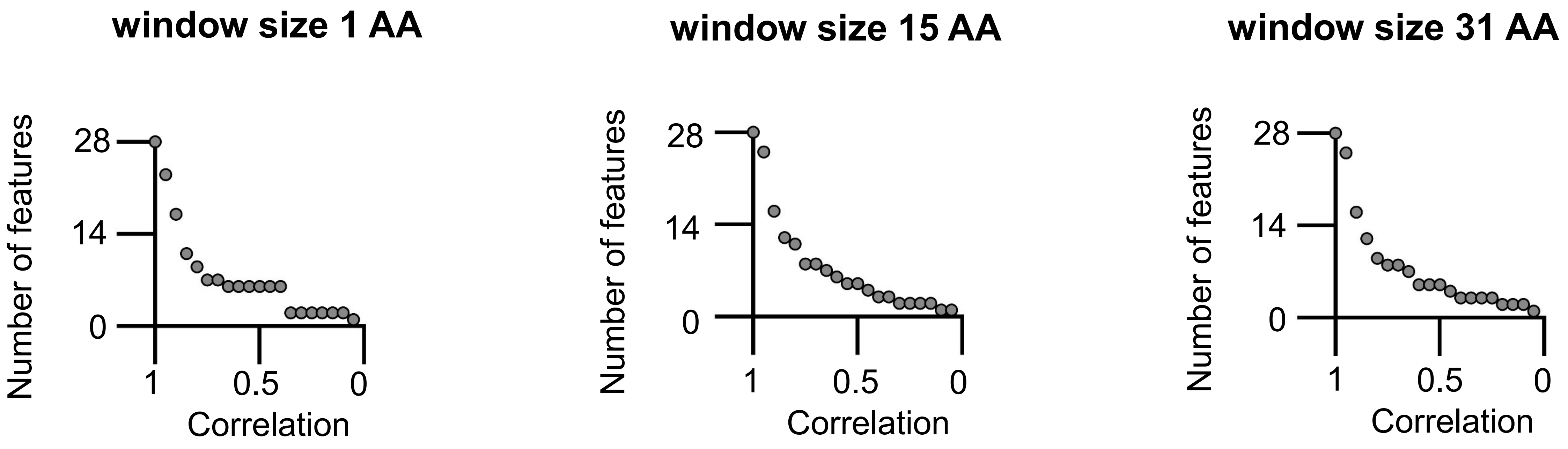

Figure S5. Correlation filter for 28 features. (A) Application of 95% correlation filter on all features at 1 AA, 15 AA and 31 AA window size MA. (B) Closer look how AA window size (20 features) influences feature correlations.

Forward feature selection

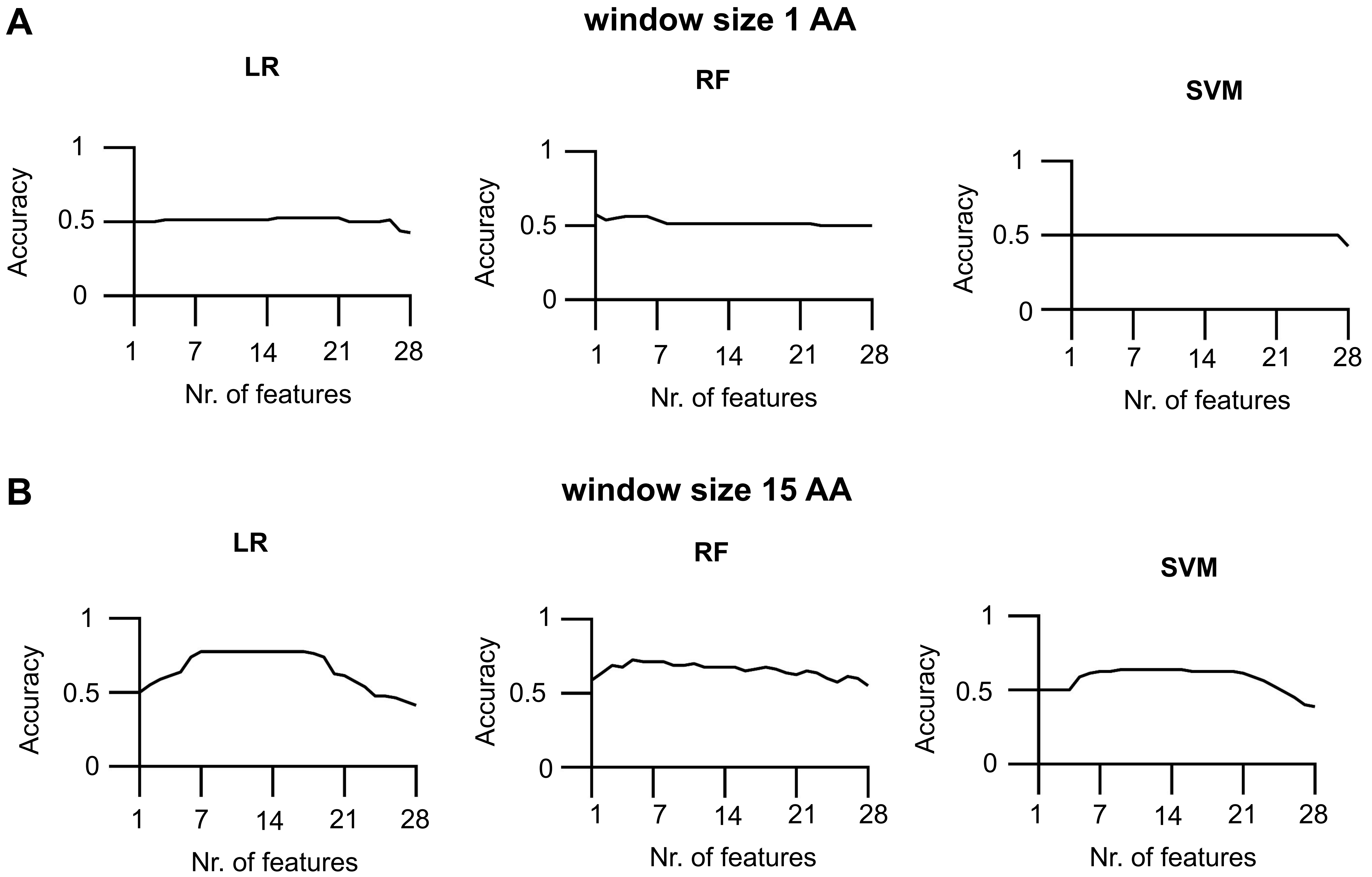

Figure S6. Forward feature selection used on three different ML methods at window size 1 AA and 15 AA. (A) In general, there is no significant improvement in the accuracy. The highest accuracy (0.575) has RF with one selected feature. (B) After application of window size 15 AA, there are significant improvements in the accuracy – in particular in the LR method (0.775) with 7 out of 17 features and RF algorithm accuracy (0.75) with 5 features. In this case, the SVM method has the lowest improvements (0.6375) with nine out to fifteen features.

cross validation LR

Table S2: 10-folds Cross-validation of the LR model on whole data set

k-folds	Error in %	Size of Test Set
[redacted]		
Fold 2	10.3448	58
[redacted]		
Fold 4	13.7931	58
[redacted]		
Fold 6	7.0175	57
[redacted]		
Fold 8	21.0526	57
[redacted]		
Fold 10	12.2807	57

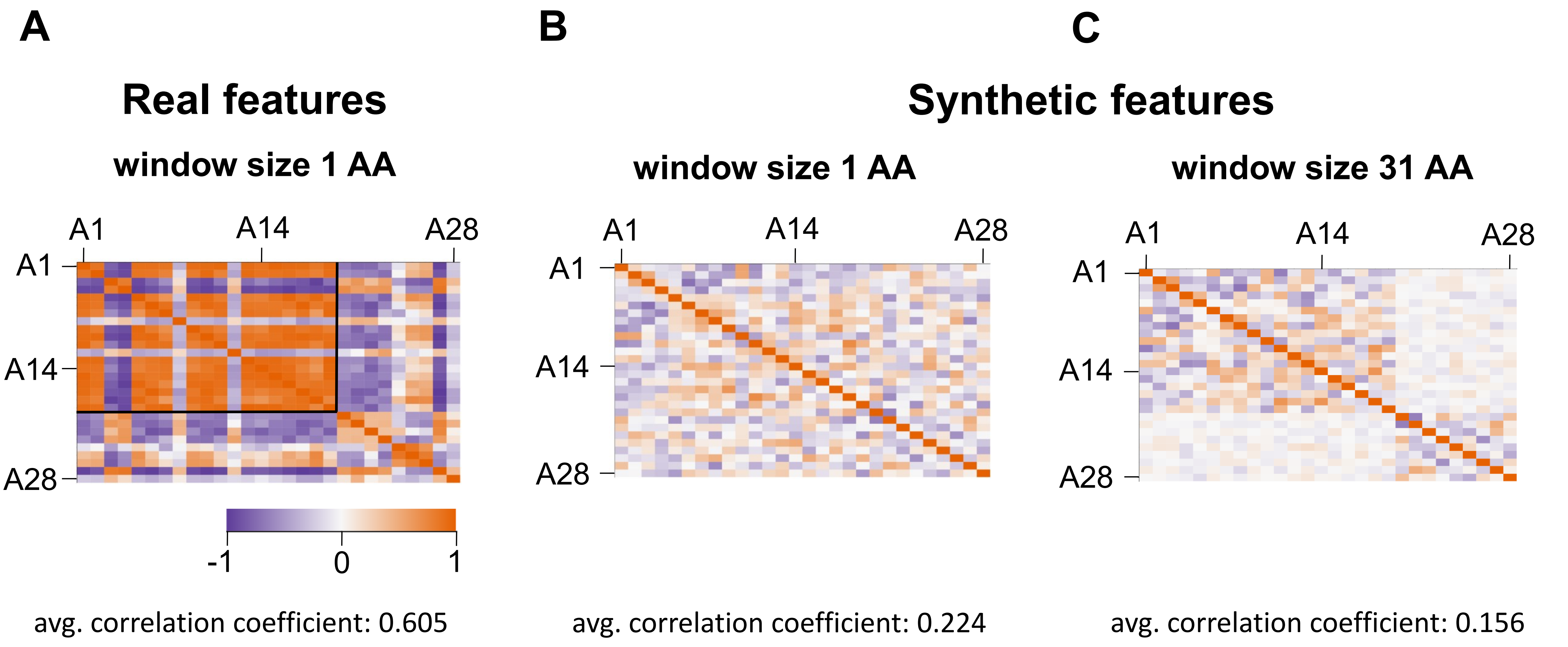

Figure S7. Heat maps of feature correlations for real feature values and for synthetic feature values at window sizes 1 AA and 31 AA respectively. (A) Heat map of real feature values at window size 1 AA shows larger average correlation coefficient (0.61) in comparison to synthetic features at window size 1 (0.22). The correlation is more significant for A1 - A20 features (MA applied) and for features A21 to A28 (no MA). (B) The average correlation coefficient is higher (0.22) for synthetic features at window size 1 AA then at window size of 31 AA (0.16). (C) Synthetic feature values at window size 31 AA. The average correlation coefficient is 0.15.

SVM – cross validation

Table S3: 10-folds Cross-validation of SVM on whole data set

k-folds	Error in %	Size of Test Set
[redacted]		
Fold 2	18.9655	58
[redacted]		
Fold 4	10.3448	58
[redacted]		
Fold 6	24.5614	57
[redacted]		
Fold 8	17.5439	57
[redacted]		
Fold 10	28.0702	57

Stdev = 5.7921

Table S4: Statistics of SVM model on whole data set.

Set (windows size 31)	Category	Recall	Precision	F-measure	Accuracy	Cohen's kappa
[redacted]						
[redacted]	S	0.9070	0.7778	0.8375		
[redacted]						

RF– cross validation

Table S5: 10-folds Cross-validation of RF on whole data set

k-folds	Error in %	Size of Test Set
[redacted]		
Fold 2	27.5862	58
[redacted]		
Fold 4	18.9655	58
[redacted]		
Fold 6	10.5263	57
[redacted]		
Fold 8	19.2982	57
[redacted]		
Fold 10	21.0526	57

Stdev = 4.3612

Table S6: Statistics of RF model on whole data set.

Set (windows size 31)	Category	Recall	Precision	F-measure	Accuracy	Cohen's kappa
[redacted]						
[redacted]	S	0.8592	0.8199	0.8391		
[redacted]						
